# The effect of topically applied tissue expanders on radial forearm skin pliability: a prospective self-controlled study

**DOI:** 10.1186/1916-0216-43-8

**Published:** 2014-04-16

**Authors:** Jeffson Chung, James P Bonaparte, Michael Odell, Martin Corsten

**Affiliations:** 1 The Department of Otolaryngology - Head & Neck Surgery, Ottawa Hospital - General Campus S3, 501 Smyth Road, Ottawa, Ontario K1H 8 L, Canada

**Keywords:** Radial forearm free flap, Tissue expansion, Cutometer

## Abstract

**Background:**

The use of pre-operatively applied topical tissue expansion tapes have previously demonstrated increased rates of primary closure of radial forearm free flap donor sites. This is associated with a reduced cost of care as well as improved cosmetic appearance of the donor site. Unfortunately, little is known about the biomechanical changes these tapes cause in the forearm skin. This study tested the hypothesis that the use of topically applied tissue expansion tapes will result in an increase in forearm skin pliability in patients undergoing radial forearm free flap surgery.

**Methods:**

Twenty-four patients scheduled for head and neck surgery requiring a radial forearm free flap were enrolled in this prospective self-controlled observational study. DynaClose tissue expansion tapes (registered Canica Design Inc, Almonte, Canada) were applied across the forearm one week pre-operatively. Immediately prior to surgery, the skin pliability of the dorsal and volar forearm sites were measured with the Cutometer MPA 580 (registered Courage-Khazaka Electronic GmbH, Cologne, Germany) on both the treatment and contralateral (control) arms. Paired t-tests were used to compare treatment to control at both sites, with p < 0.025 defined as statistically significant.

**Results:**

There was a statistically significant increase in pliability by a mean of 0.05 mm (SD = 0.09 mm) between treatment and control arms on the dorsal site (95% CI [0.01, 0.08], p = 0.018). This corresponded to an 8% increase in pliability. In contrast, the volar site did not show a statistically significant difference between treatment and control (mean difference = 0.04 mm, SD = 0.20 mm, 95% CI [−0.04, 0.12], p = 0.30).

**Conclusions:**

This result provides evidence that the pre-operative application of topical tissue expansion tapes produces measurable changes in skin biomechanical properties. The location of this change on the dorsal forearm is consistent with the method of tape application. While this increase in skin pliability may account for the improved rate of primary donor site closure reported using this technique, the results did not reach our definition of clinical significance.

## Background

The radial forearm free flap is a common and versatile reconstructive option in head and neck cancer [1]. Until recently, one disadvantage was that the donor forearm was left with a defect that had a low rate of primary closure. Traditionally, this defect required a split thickness skin graft harvested from the thigh, which resulted in additional morbidity [2-4].

**Figure 1:**
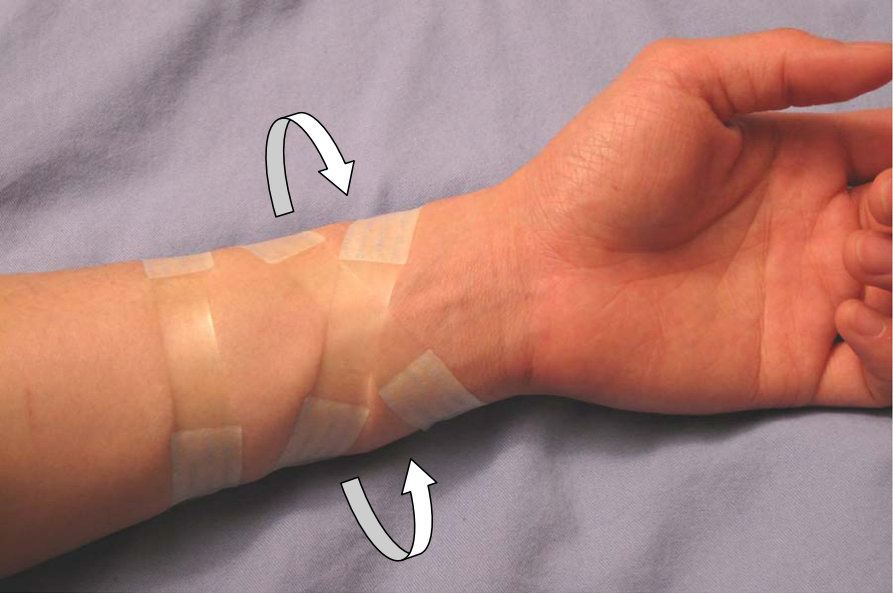
**Tension tapes on the treatment arm.** Typical application of tension tapes on the planned donor arm. Note the transverse proximal tape and the crisscross arrangement of the two distal tapes. Also note that the elastic portion of the tape overlies the volar aspect of the forearm. Arrows show direction of force exerted by the elastic.

In 2007, the head and neck surgeons at our centre sought to improve primary closure rates in the forearm by using DynaClose tension tapes (registered Canica Design Inc, Almonte, Canada) as a new method of donor site management. These tapes were composed of two adhesive ends joined by an elastic that applied a persistent linear stretching force (Figure 1), thereby producing tissue expansion pre-operatively [5,6]. In a recent series of 177 patients treated with this tape, we avoided a thigh donor site in 95% of forearm defects as they were closed either primarily or by using a small full thickness skin graft harvested from redundant tissue on the ipsilateral arm incision line [7]. This method of donor site management reduced the need for split thickness skin grafts, which resulted in improved cosmetics, reduced pain and a reduced economic cost of wound care [8,9].

While studies have shown that these tapes result in improved surgical outcomes, several limitations remain. All studies published on this technique thus far were either unblinded trials or large case series [7-9]. Outcome measures for these studies were clinical and often relied on subjective measures. In addition, it was unclear if the tissue expansion tapes were responsible for the increase in primary closure rates or if other factors were responsible. Finally, there was a paucity of data on the effect of these tapes on forearm skin biomechanics. Thus, we designed a study to investigate this using the Cutometer MPA 580 (registered Courage-Khazaka Electronic GmbH, Cologne, Germany). The Cutometer is a non-invasive instrument capable of reliably measuring the skin’s biomechanical properties in vivo [10-12]. It consists of a probe which applies a suction load when applied against the skin. The resulting skin deformation is then measured and plotted as a function of time, and various skin properties derived from the loading-unloading curve. Specifically, we chose to examine pliability (Uf), which was shown by Nedelec et al. to be a highly reliable study parameter for skin extensibility (intraclass correlation coefficient > 0.89) [11]. Gaining a better understanding of the tissue expansion tapes’ effect on skin pliability can be valuable in future attempts to optimize their efficacy. We may even be able use this knowledge to better predict which patient group would benefit from this device and which group would most likely achieve full primary closure of the their radial forearm wounds.

The nature of the DynaClose tapes combined with our method of application is such that the maximal stretch on the forearm skin is on the dorsal surface, with minimal stretch on the volar aspect. The purpose of this study was to test the hypothesis that a one week application of DynaClose tapes would result in an increase in forearm skin pliability at the dorsal forearm site but not at the volar forearm site.

## Methods

### Subjects

All head and neck cancer patients scheduled for a radial forearm free flap during the study period (October 2011 – September 2012) were prospectively enrolled. Patients were excluded if they met any of the following exclusion criteria:

•Patients who did not receive pre-operative tissue expansion due to the emergency nature of their surgery, and

•Patients whose tissue expansion tapes fell off more than 8 hours prior to surgery.

Twenty four patients met inclusion criteria and none declined to participate. All patients received the intervention and were included in the analysis. Table 1 summarizes the patient demographics. Each patient’s non-donor arm served as their own control. To confirm this was valid, our team conducted a pilot study with 30 patients and 2 observers demonstrating that at baseline there is no significant difference in skin pliability between a patient’s dominant and non-dominant forearms (95% confidence interval for the mean difference in pliability overlaps zero, and p > > 0.05 for both the dorsal and volar sites) [13]. This strategy also allowed us to control for inherent variability in skin between patients due to factors such as age and sex.

**Table 1:** Patient characteristics

**Number of patients N**	**24**
Mean age	64
Minimum age	46
Maximum age	83
Standard deviation	12
Male: Female	11 : 13
Location of primary cancer (%)	
Tongue	12 (50%)
Floor of mouth	3 (13%)
Retromolar trigone	2 (8%)
Larynx	2 (8%)
Oropharynx	2 (8%)
Alveolar ridge	1 (4%)
Palate/maxilla	1 (4%)
Sinonasal	1 (4%)
Total	24 (100%)

Summary of patient characteristics.

All patients signed a consent form prior to their inclusion. This study was approved by the Ottawa Hospital Research Ethics Boards under protocol #2009622-01H.

### Experimental protocol

In all cases, the intervention was the application of topical tissue expansion tapes, which was performed in clinic one week pre-operatively. The tapes were applied on the planned donor arm as shown in Figure 1. Two distal tapes were applied in a crisscross fashion with a third proximal tape in a transverse orientation, all with the elastic portion on the volar aspect. No forearm was shaved prior to tape application. Patients were instructed to return to the clinic if their tapes fell off at any point prior to surgery for reapplication. Those who did not present within 8 hours for reapplication were excluded from the study. Immediately before surgery, the tapes were removed and skin cleaned with alcohol swabs prior to measurement of both forearms with the Cutometer MPA 580 (registered Courage-Khazaka Electronic GmbH, Cologne, Germany).

All subjects were recruited on the morning of their surgery, which was deemed appropriate because the application of tissue expansion tapes was standard procedure at our institution; all head and neck surgery patients requiring radial forearm free flaps received this intervention one week prior to surgery regardless of participation in this study. Consent to participate in the study also did not affect randomization because each patient served as their own control. Thus, consenting to the study did not affect patient care in any way.

**Figure 2:**
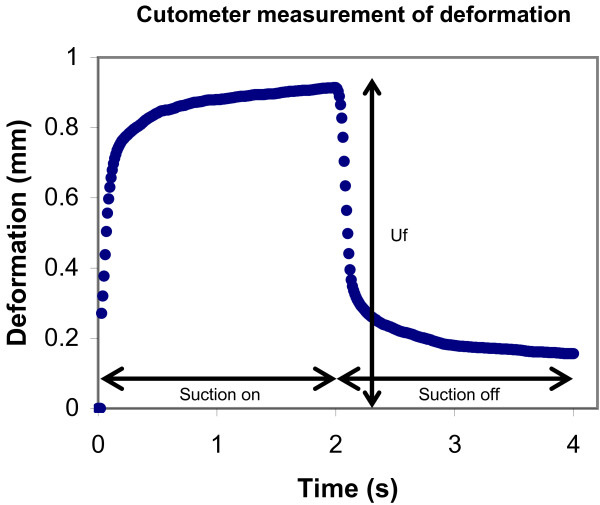
**Cutometer loading and unloading curve.** Typical deformation curve of skin under one cycle of loading and unloading. The Cutometer measures deformation of skin in millimeters as a function of time in seconds. Here, 45 kPa of suction was applied for 2 seconds, followed by no suction for 2 seconds. Uf is labeled and represents the maximal amount of skin deformation due to the suction load.

The Cutometer MPA 580 applies a 45 kPa suction through a 6 mm aperture in the probe and measures the vertical deformation of the skin as a function of time. One cycle comprises of a constant suction for two seconds, followed by no suction for two seconds. A typical deformation curve is shown in Figure 2. For our study, we examined Uf - the maximal deformation under the suction load. Uf was measured in millimeters, and represented a measure of pliability, not the total gain in expanded tissue. Measurements were performed 6 cm proximal to the wrist crease on both the volar and dorsal aspects of the pre-expanded donor forearm and the control forearm. Each side of the forearm was measured three times and the average for Uf was taken. In between each measurement, the probe was lifted off the skin and the machine allowed to recalibrate to 45 kPa. The probe was then repositioned on a closely adjacent, previously untested patch of skin.

**Figure 3:**
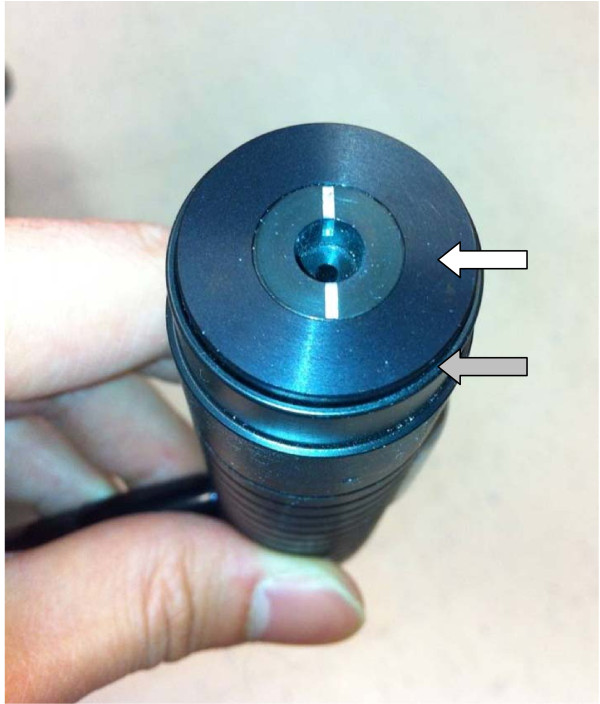
**Cutometer probe.** The Cutometer probe viewed end on. The white arrow shows the spring mounted contact surface which contains the 6 mm aperture. The grey arrow shows the rigid external shell that is held by the operator.

The probe itself is composed of an external cylindrical shell that is held by the operator. Protruding just beyond the end of the shell is a spring mounted contact surface containing the 6 mm aperture (see Figure 3). Prior to measurement, a piece of double sided tape was placed around the aperture. This prevented inadvertent movement of the probe during measurements. Just enough pressure was applied so that the external shell was flush with the contact surface and the patient’s skin. This standardized the contact pressure and minimized the effect of hand tremor on the measurements.

All measurements were taken by authors JC and JPB who were experienced users of the Cutometer.

### Sample size calculation

We estimated our sample size for a two-sided paired *t*-test with alpha = 0.025 and power of 80%. Based on pilot testing, we used a standard deviation in pliability of 0.15 mm, which resulted in a sample size of 24 [13]. A 10% change in pliability was arbitrarily defined as clinically significant as no previous literature on what constitutes clinically significant change in pliability as measured by the Cutometer exists. The Anderson-Darling test was used to ensure a normal distribution of the skin parameter Uf (p > > 0.05). The paired *t*-test was used as a test for significance with p < 0.025 defined as a statistically significant difference (Bonferroni correction factor of 2 for two forearm sites). Data analysis was carried out using Minitab 15.

## Results

**Figure 4:**
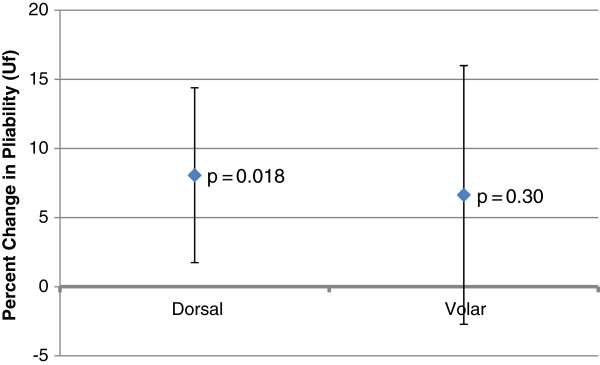
**Mean difference in pliability.** Mean difference in pliability between treatment and control arms for both dorsal and volar sites. The bars indicate 95% confidence intervals.

For the dorsal aspect of the forearm, the mean difference in pliability between the treatment and control arms of each patient was +0.05 mm (SD = 0.09 mm); the 95% confidence interval was [0.01, 0.08 mm]. See Table 2. This corresponded to a mean increase in pliability of 8% (SD = 16%), which reached statistical significance (p = 0.018). See Figure 4.

**Table 2:** Dorsal forearm cutometer measurements

**Patient**	**Uf of control forearm (mm)**	**Uf of treatment forearm (mm)**	**Treatment-control (mm)**	**% Change**
1	0.86	1.08	0.22	25.19
2	0.59	0.67	0.08	13.06
3	0.88	0.94	0.07	7.77
4	0.89	0.84	−0.06	−6.42
5	0.78	0.92	0.14	18.26
6	0.69	0.72	0.03	3.73
7	0.76	0.70	−0.06	−8.09
8	0.81	0.73	−0.08	−10.25
9	0.58	0.61	0.03	5.50
10	0.62	0.67	0.05	8.00
11	0.87	0.86	0.00	−0.54
12	0.54	0.69	0.15	27.99
13	0.65	0.64	−0.01	−1.34
14	0.59	0.56	−0.03	−5.51
15	0.78	0.79	0.01	0.93
16	0.72	0.79	0.07	9.42
17	0.70	0.72	0.02	3.36
18	0.53	0.51	−0.02	−3.28
19	0.63	0.62	−0.01	−0.96
20	0.64	0.77	0.14	21.87
21	0.60	0.58	−0.02	−3.41
22	0.48	0.60	0.12	24.08
23	0.48	0.78	0.30	61.51
24	0.72	0.74	0.02	2.76
Mean			0.05	8%
95% Confidence interval			0.04	6%
2 tailed paired *t* test p = 0.018				

For the volar aspect of the forearm, the mean difference in pliability between the treatment and control arms of each patient was +0.04 mm (SD = 0.20 mm); the 95% confidence interval was [−0.04, 0.12 mm], which overlapped zero. See Table 3. This mean difference in pliability between the treatment and control arms at the volar site was 7% (SD = 23%) but did not reach statistical significance (p = 0.30). See Figure 4.

**Table 3:** Volar forearm cutometer measurements

**Patient**	**Uf of control forearm (mm)**	**Uf of treatment forearm (mm)**	**Treatment-control (mm)**	**% Change**
1	1.16	1.32	0.16	13.36
2	0.84	0.93	0.09	10.74
3	1.39	1.44	0.05	3.49
4	1.60	1.36	−0.24	−14.73
5	0.99	1.28	0.29	29.59
6	1.35	1.08	−0.27	−20.19
7	0.90	0.87	−0.02	−2.38
8	0.79	0.61	−0.18	−23.27
9	0.76	0.66	−0.11	−13.98
10	0.82	1.14	0.33	39.87
11	0.78	1.08	0.30	38.31
12	0.56	0.57	0.01	2.15
13	0.64	0.87	0.23	35.21
14	0.76	1.06	0.30	39.42
15	1.01	1.08	0.07	7.33
16	0.74	1.07	0.34	45.42
17	0.90	0.73	−0.17	−18.69
18	0.85	0.70	−0.15	−18.00
19	0.79	1.04	0.25	32.23
20	1.11	0.97	−0.14	−12.87
21	0.66	0.63	−0.03	−3.95
22	0.83	0.63	−0.20	−24.27
23	0.95	0.87	−0.09	−8.97
24	1.00	1.23	0.24	23.61
Mean			0.04	7%
95% Confidence interval			0.08	9%
2 tailed paired *t* test p = 0.30				

## Discussion

We believe that DynaClose tapes produce mechanical creep in the treatment arm which translates to decreased tension in wound closure [14] and increased rate of primary closure with or without a small full thickness skin graft [7]. Our hypothesis was that there would be an observable change in pliability, measured here as an increased vertical deformation under a given suction load. Our results showed that there was a statistically significant difference in skin pliability between the pre-expanded donor forearm and the control forearm on the dorsal aspect. This is in support of our hypothesis that tissue expansion using topically applied tapes increases skin pliability. However, the change in pliability did not reach our definition of clinical significance, which was based on our own pilot testing as no previous data exists on this subject. Our pilot study of 30 patients comparing each patient’s two untreated forearms showed a difference in pliability of 4% based purely on chance [13]. It was for this reason that a threshold of 10% was chosen for a clinically significant change. However, what a 10% change in pliability means in terms of absolute tissue gain is currently unknown. There is certainly room for future investigations to better understand how changes in pliability translate to tissue expansion.

One of the previously unknown factors regarding this topically applied tissue expansion technique was where the tension was actually being applied to the skin. By measuring changes in pliability at multiple sites on the arm, our goal was to identify the location at which the skin was being influenced. These results showed that the tapes produced a change in pliability on the dorsal aspect of the forearm but not the volar aspect, suggesting that the effect was centered at the dorsal forearm. This was consistent with how the tapes work and how they were applied. The linear stretch forces from the tape were transferred to the skin through the adhesive ends, which were attached to the dorsal forearm. As the elastic relaxes and shortens, the adhesive ends stretch the dorsal forearm skin and pull it towards the volar aspect (see Figure 1). Interestingly, the absolute changes in pliability of the dorsal and volar forearms turned out to be very similar (8% vs. 7%) though only the change in the dorsal forearm reached statistical significance. This was due to the fact that the volar forearm exhibited a large variability in pliability, which ultimately resulted in a larger 95% confidence interval that overlapped zero, as well as a larger p-value on the paired *t*-test. The reason for this is uncertain, though the biomechanical characteristics of the thin hairless volar skin are expectedly different from the thicker hair-bearing dorsal skin. Thus, a direct comparison between dorsal and volar forearm skin may not be valid, i.e. the treatment dorsal forearm can only be compared with the control dorsal forearm, and the treatment volar forearm can only be compared with the control volar forearm.

Another possible reason our measured change in pliability didn’t reach clinical significance may be due to our study design. We designed our study to compare the non-expanded, non-donor arm to the expanded donor arm because it allowed the measurements to be taken by a single operator at a single sitting, rather than over two sittings, possibly by different operators. This precluded any possibility of losing patients to follow up, and inter-rater differences in measuring technique were minimized. This latter point was important in light of the fact that differences in contact pressure between the Cutometer probe and the skin can affect measurements [15]. Furthermore, we have shown in a pilot study that there was no difference in pliability between a patient’s two arms at baseline [13]. In other words, one should be able to use the non-donor arm to represent the state of the donor arm prior to expansion, and interpret any measured differences as being a direct result of tissue expansion. However, to measure the effect of topically applied tissue expansion tapes on the skin properties of the donor forearm, one would ideally compare the non-expanded state to the expanded state in the same arm, 1 week apart. This would more clearly demonstrate the effect of the tapes, rather than have it partly obscured by chance differences between the forearms.

Another reason why the measured change in pliability fell short of the defined threshold for clinical significance may be due to the method of measuring pliability. The Cutometer essentially performs the pinch test – an informal predictor of the ease of wound closure [16] – in an objective way. However, it is capable of measuring some nine different parameters and deriving four different ratios to describe the viscoelastic nature of skin [17,18]. Exactly which parameter is the most representative of qualities such as elasticity and pliability is currently unknown. In the existing literature, many of these parameters have been studied and reported on, though the choice depended on the application [19-23]. The parameter chosen for our study, Uf, was thought to be a suitable one as it was a measure of both the elastic and viscous properties of skin and represents the mechanical creep induced by tissue expansion. Furthermore, it was found to be highly reliable by Nedelec et al. [11]. Perhaps a different parameter may show a bigger effect from the expansion tapes. It is clear, however, more research needs to be done to study the Cutometer as an investigative tool. A potential future study would be to see how changes in pliability correspond to rate of primary closure, thus evaluating the clinical significance of these changes. Undoubtedly flap size and patient age will also play a factor.

The DynaClose tension tape system is relatively non-invasive compared to other methods of tissue expansion and has few adverse effects. In our series, the only noted adverse event was a mild self-limited skin reaction to the adhesive in some of the patients, none of which resulted in discontinuation of the tapes. A notable shortcoming in the DynaClose tape is that the adhesive can occasionally fail and come undone. This is especially the case in males with hairy forearms. When this happens, patients are instructed to return to clinic for reapplication, but not all patients do and they were excluded from this study. One possibility for reducing the loss of adhesion would be to shave the donor forearm prior to tape application. Another method would be to reinforce the adhesive ends with additional tape. Overall, however, our success rate in maintaining the tapes (92%) and achieving tissue expansion remains very high [7]. Future studies into this topically-applied two-dimensional expansion system can evaluate other methods of applying the tapes. For example, one can see whether applying more tapes results in further increases in pliability. In the later phase of tissue expansion, stress relaxation also occurs through increased mitotic activity and new tissue formation in a process termed biologic creep [24]. This stress relaxation would most likely decrease the efficacy of the tapes over time. Thus, it would be interesting to see if reapplication of the tapes at regular time intervals can also result in further increases in pliability and tissue expansion.

## Conclusions

We have demonstrated that topically applied tissue expanders create tissue expansion by measuring a statistically significant change in forearm skin pliability. The change occurs on the dorsal aspect of the forearm, which is consistent with the method of tape application. Though this increase in skin pliability did not reach our definition of clinical significance, we believe it may account for the improved rate of primary donor site closure reported earlier using this technique. The Cutometer proved to be a reliable instrument that can be useful for future research into topically applied tissue expansion tapes.

## Abbreviations

SD: Standard deviation; CI: Confidence interval; Uf: Maximal deformation of skin under a given suction load, defined here as pliability.

## Competing interests

The authors declare that they have no competing interests.

## Authors’ contributions

JC participated in the design of the study, recruitment of patients, collection of data, and drafted the manuscript. JPB conceived of the study, participated in the design of the study, recruitment of patients, collection of data, and performed the statistical analysis. MO participated in the recruitment of patients. MC participated in the recruitment of patients, and editing of the manuscript. All authors read and approved the final manuscript.
